# Toward equitable major histocompatibility complex binding predictions

**DOI:** 10.1073/pnas.2405106122

**Published:** 2025-02-18

**Authors:** Eric Glynn, Dario Ghersi, Mona Singh

**Affiliations:** ^a^Department of Molecular Biology, Princeton University, Princeton, NJ 08544; ^b^Lewis-Sigler Institute for Integrative Genomics, Princeton University, Princeton, NJ 08544; ^c^School of Interdisciplinary Informatics, University of Nebraska at Omaha, Omaha, NE 68182; ^d^Department of Computer Science, Princeton University, Princeton, NJ 08544

**Keywords:** predicting major histocompatibility complex (MHC) binding, health equity in precision oncology, neoantigen prediction, cancer immunotherapies, deep learning

## Abstract

Deep learning tools that predict peptide binding by major histocompatibility complex (MHC) proteins play an essential role in developing personalized cancer immunotherapies and vaccines. In order to ensure equitable health outcomes from their application, MHC binding prediction methods must work well across the vast landscape of MHC alleles observed across human populations. Here, we show that there are alarming disparities across individuals in different racial and ethnic groups in how much binding data are associated with their MHC alleles. We introduce a machine learning framework to assess the impact of this data imbalance for predicting binding for any given MHC allele, and apply it to develop a state-of-the-art MHC binding prediction model that additionally provides per-allele performance estimates. We demonstrate that our MHC binding model successfully mitigates much of the data disparities observed across racial groups. To address remaining inequities, we devise an algorithmic strategy for targeted data collection. Our work lays the foundation for further development of equitable MHC binding models for use in personalized immunotherapies.

## Significance

A critical component of designing immunotherapies tailored to individual patients is to identify cancer mutations that their immune systems can recognize as foreign. Major histocompatibility complex (MHC) proteins perform this recognition via physical interactions, and machine learning methods are frequently employed to predict MHC binding. However, there are thousands of MHC variants, and binding data are known for only a small fraction of them. Training on these data can result in machine learning models that have disparate performance across MHC variants and, therefore, across individuals. To address this, we introduce computational techniques that quantify and mitigate the impact of data bias on MHC binding predictions. Our work is an important step toward developing equitable computational approaches for personalized immunotherapies.

Immunotherapies, where individuals’ immune systems are harnessed to attack cancer cells, are among the most exciting new approaches for cancer treatment (1). T cells, which are central components of this process, detect and then either eliminate cells that display foreign or mutated peptides in complex with major histocompatibility complex (MHC) molecules on their surfaces or secrete signals to stimulate other immune cells. As MHC proteins are highly polymorphic across human populations and each allele can bind a different set of peptides (2), machine learning methods to predict which peptides each MHC protein can bind (3) have become essential in screening cancer proteomes for potential T cell epitopes (4, 5).

In silico T cell epitope screens utilize MHC–peptide binding predictions to identify cancer neoantigens, which are peptides generated from tumor-specific mutations that are presented by an individual’s MHC molecules. Landmark advances in cancer immunotherapy, brought about with checkpoint blockade inhibitors, have resulted in therapeutics that can overcome the multiple escape mechanisms that tumors can use to evade T cell–mediated elimination (5). These advances have propelled neoantigen discovery and the development of personalized neoantigen vaccines–numerous of which are in clinical trials–to therapeutically target tumors with a level of specificity unrivaled by existing cancer treatments (6, 7). The improvement of computational methods for predicting MHC–peptide binding is frequently cited as one of the greatest limitations, and areas of need, for the efficacy of emergent neoantigen immunotherapies (8).

A major challenge in predicting MHC–peptide binding is that while there are over 13,000 distinct class I MHC protein allelic variants (9), binding data are only available for a relatively small number of MHC alleles. Pan-MHC models take as inputs representations of an MHC allele and a peptide sequence and leverage the similarities among MHC alleles and their binding repertoires in order to make binding predictions for any MHC allele (10–16). However, a major limitation of current approaches is that predictive performance can only be estimated for the small minority of MHC alleles with sufficiently large binding datasets. Since MHC binding algorithms influence the efficacy of downstream biotechnologies (8, 17, 18), it is absolutely necessary to understand the quality of MHC–peptide predictions for any allele. Moreover, because the frequency of MHC alleles varies across different human subpopulations (19), there is substantial danger that differing MHC–peptide prediction performance across alleles may yield disparate outcomes in immunotherapies across racial and ethnic groups, thereby exacerbating the already significant disparities in cancer mortality and treatment across racial groups (20). Existing approaches to predict MHC–peptide binding (21) have not been designed or comprehensively evaluated with respect to equitable performance across individuals and populations.

Here, we introduce a framework to enable the development of equitable class I MHC binding prediction models. We begin by highlighting the importance of developing MHC binding models with the explicit goal of equitable predictions by demonstrating that there are stark disparities in how much binding data is associated with the MHC alleles of different individuals and of individuals across racial and ethnic groups. We next introduce a framework for equitable MHC models based upon the codevelopment of a deep learning model MHCGlobe for predicting peptide–MHC binding, along with a machine learning method MHCPerf to estimate how well this pan-MHC model performs on unseen MHC alleles. We demonstrate that MHCGlobe has state-of-the-art performance in predicting MHC–peptide binding as well as high estimated performance for the majority of MHC alleles, despite most of them having little or no binding data. These performance estimates across all alleles—even those with no existing binding data—comprise an analysis of how well pan-MHC models support the diversity of MHC alleles found across human populations. Despite excellent overall per-allele performance, we find that MHCGlobe nevertheless exhibits some performance differences across MHC alleles and this leads to disparate performance estimates when applied to individuals from different racial groups. Finally, we devise algorithms to select MHC alleles to prioritize for future data collection as a strategy to address the effect of data imbalances on MHC binding model performance.

The current study contributes multiple resources to clinicians, immunologists, and computational researchers whose work intersects with the adaptive immune response to MHC-bound antigens. For personalized vaccine design, our allele-level performance estimates can be used to prioritize neoantigens for which predictions are expected to be of higher quality. For experimentalists, we prioritize MHC alleles for which data collection is estimated to have great utility in improving equitable performance across the landscape of underserved alleles. For researchers actively working to improve performance of machine learning models for MHC binding, we release the most comprehensive assessment of how the performance of a pan-MHC model is affected by data collection, including allele-level performance estimates revealing model “blind spots” that need to be addressed. Further, ethical questions pertaining to individual and group fairness are raised here and invite further work to establish metrics of fairness in the field of T cell antigen discovery and personalized therapies. Individually and combined, we hope these resources inspire future efforts to measure and mitigate inequality in MHC binding models and in T cell epitope discovery as well as to promote innovations to ensure both effective and equitable personalized therapies.

## Results

### Amount of Binding Data Across Human MHC Class I Alleles Varies Significantly Across Individuals and Racial and Ethnic Groups.

**Fig. 1.**
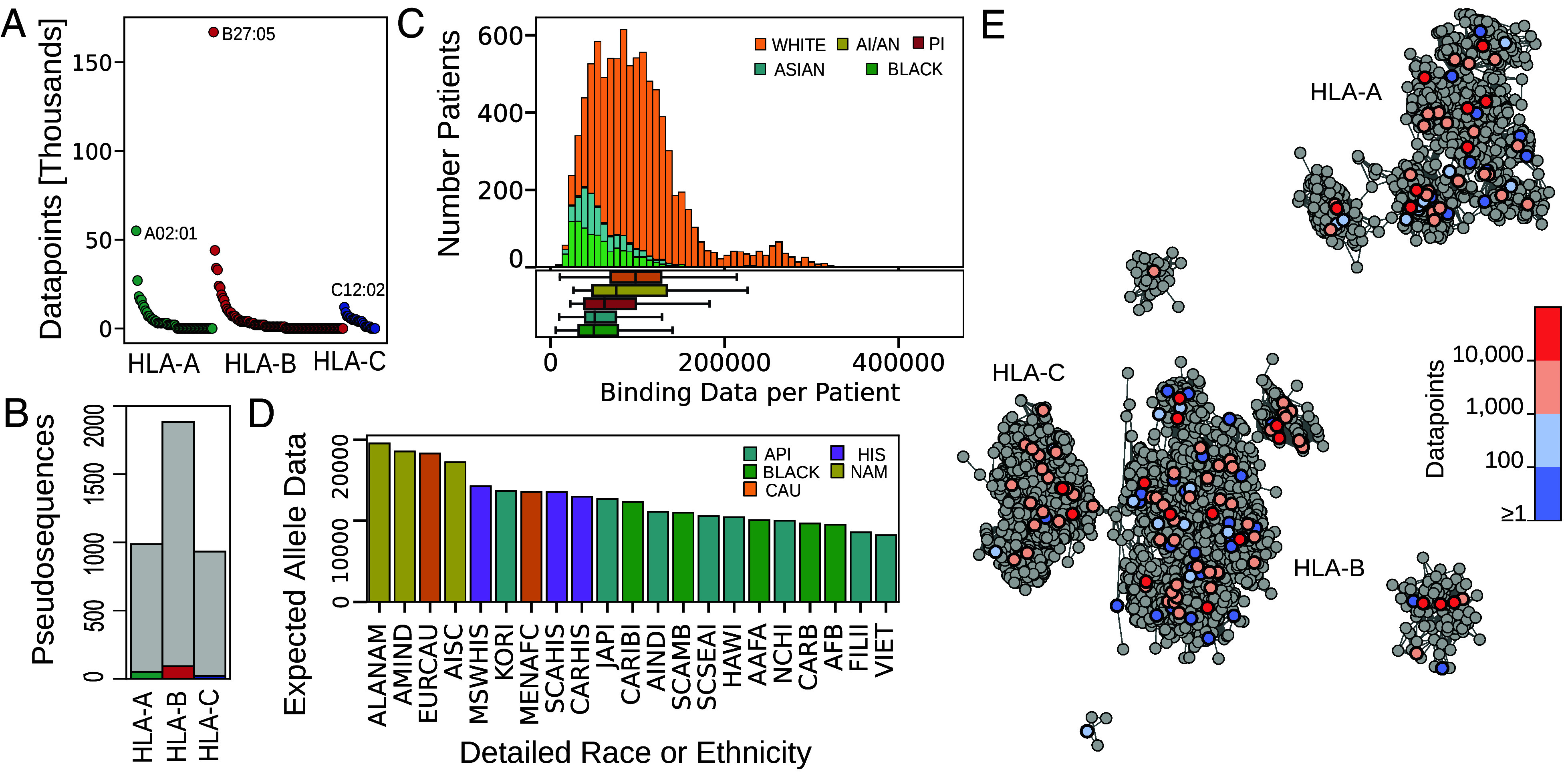
Variation in peptide–MHC binding data per HLA allele and across racial and ethnic groups. (*A*) The number of positive peptide–MHC binding datapoints for each of the 180 HLA-A, HLA-B, or HLA-C alleles with any data; each dot represents one allele. The allele with the most data for a given gene is labeled. (*B*) The number of distinct HLA pseudosequences with binding data (colored) or without (gray) per HLA gene. (*C*) (*Top*) A histogram of the total number of datapoints associated with the class I HLAs of each of the individuals from the TCGA, computed as the sum of the number of binding peptides known for each of their six class I HLA alleles. Colors represent the race categorization recorded for each patient by TCGA (AI/AN is “American Indian or Alaska Native”, Black is “Black or African American”, and PI is “Native Hawaiian or Other Pacific Islander”). (*Bottom*) Boxplots of the total amount of data per patient, separated by race. White individuals tend to have more data associated with their MHC alleles than do Black, Asian, and Native Hawaiian or Pacific Islander individuals (*P*-values of 3.93e-196, 1.15e-141, and 0.021, respectively, two-sided Mann–Whitney test). The differences between White and AI/AN individuals are not statistically significant. (*D*) For each of 21 racial groups (see *SI Appendix*, Table S1 for descriptions), we give the expected number of positive binding instances for an allele sampled from that population according to the frequency with which that allele occurs in the population. Colors represent the broad race and ethnic categorization associated with each of the more detailed race or ethnic categorizations [Native American (NAM) in gold, Caucasian (CAU) in orange, Hispanic (HIS) in purple, Asian and Pacific Islander (API) in blue, and Black in green]. (*E*) Network visualization of 3,804 distinct MHC pseudosequences, where a node represents an individual MHC pseudosequence, and edges connect similar pseudosequences (*Materials and Methods*). Pseudosequences without associated binding data are gray, and those with data are colored according to how much data are associated with them.

In order to address how well pan-MHC binding models may be expected to work across the landscape of documented human MHC class I alleles, we first assess the binding data available for pan-MHC models to learn MHC binding patterns from. Of the greater than 13,000 documented human MHC class I protein allelic variants (HLA-A, HLA-B, and HLA-C genes), only 180 have any binding data [with 940,238 positive binding instances, and 1,119,627 total instances across all alleles (*Materials and Methods*)]. The number of positive binding instances ranges from one to over 160,000 per allele (Fig. 1*A*). Pan-MHC models commonly represent each MHC allele with a pseudosequence composed of 34 peptide-interacting residues within the MHC’s peptide binding pocket, yet only a small fraction of the distinct set of pseudosequences are associated with any binding data, corresponding to 5.56%, 5.14%, and 2.41% of HLA-A, HLA-B, and HLA-C pseudosequences, respectively (Fig. 1*B*).

We next quantify the number of positive binding instances associated with the MHC class I alleles of patients from The Cancer Genome Atlas (TCGA) project. We find highly variable coverage across individuals profiled in TCGA—ranging from 5,783 to 452,277 binding instances per patient, with a median of approximately 89,000 instances (Fig. 1*C*). By recorded race, there are statistically significant differences in the amount of data associated with the MHC alleles of White individuals as compared to Black, Asian, and Native Hawaiian or Pacific Islander individuals. To extend this analysis, we next utilize MHC allele frequencies for 21 racial and ethnic populations [estimated from HLA typing of 2.90 million individuals (19)] to calculate the expected amount of binding data for an allele sampled from each population (*Materials and Methods*). Corroborating the pattern observed in TCGA patient coverage, Caucasian and NAM populations are expected to have more data associated with a randomly sampled MHC allele as compared to Hispanic, Asian, and Black populations (Fig. 1*D*). As pan-MHC machine learning models share data across alleles in order to make predictions for alleles with limited or no binding data, we next visualize data coverage across alleles in the context of their relationships to one another using a network (Fig. 1*E*) where MHC alleles that are sequence-similar to each other are connected by an edge (*Materials and Methods*). We find that 94.3%, 99.4%, and 99.7% of MHC alleles are, respectively, adjacent to, one hop away from and two hops away from an MHC allele with data; this suggests that pan-MHC models should be able to leverage data across alleles to make good predictions for all alleles.

### Framework to Assess Allele-Level Performance of Pan-MHC Models.

**Fig. 2.**
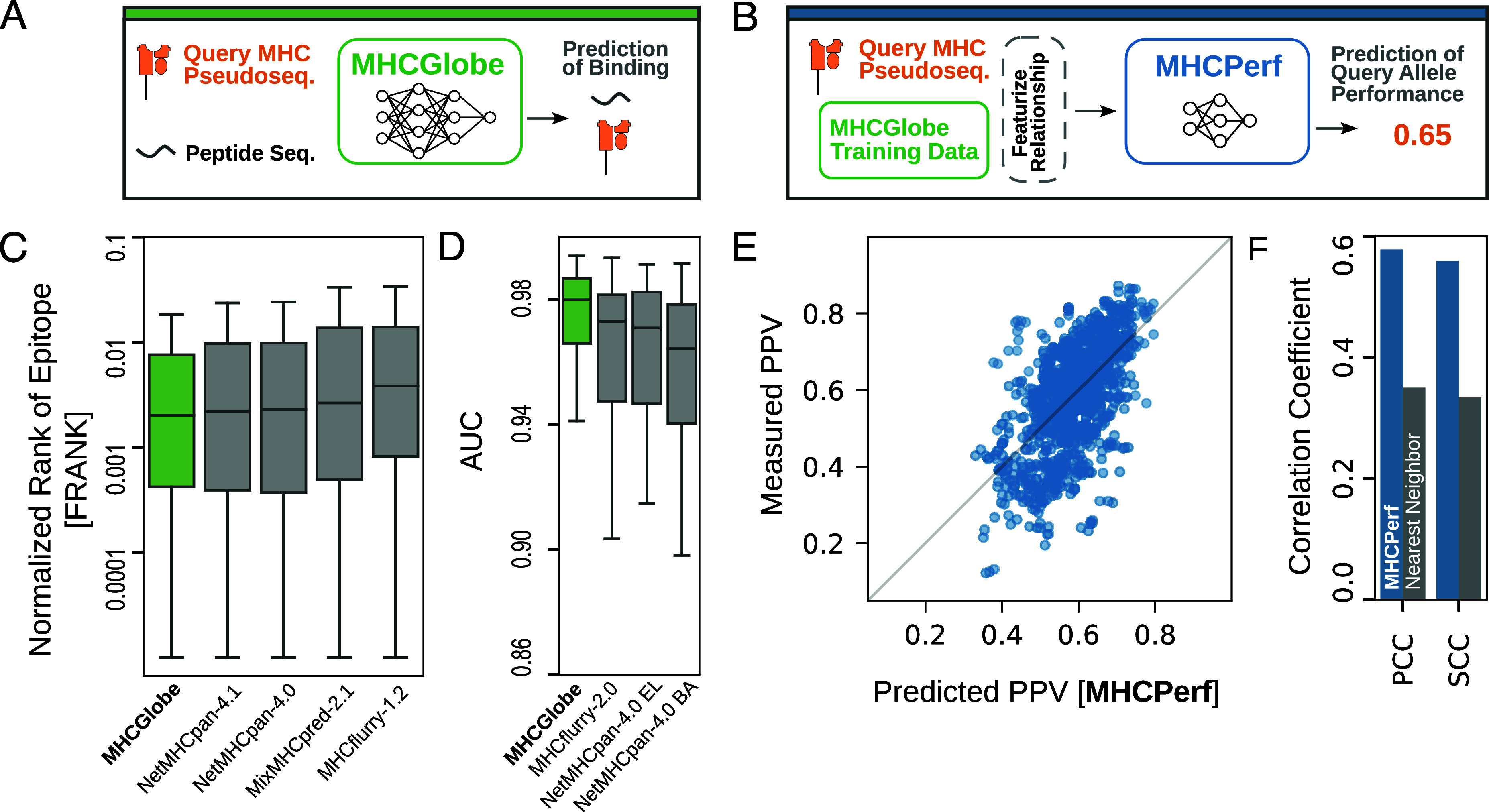
Schematic of methods and performance summary. (*A*) MHCGlobe is a pan-MHC model that predicts the BA value for an input MHC–peptide pair. (*B*) MHCPerf predicts an expected performance value (using PPV as the performance metric) that MHCGlobe would achieve for any MHC allele and specific MHCGlobe training dataset (*Materials and Methods*). (*C*) Comparison of MHCGlobe to other pan-MHC methods when evaluated on the FRANK benchmark dataset of documented CD8+ T-cell epitopes published with NetMHCpan-4.1 (Reynisson 2020). Lower FRANK values indicate that the documented epitope was ranked as a stronger MHC binder compared to other peptides from the same source protein (*Materials and Methods*). MHCGlobe has lower median FRANK scores for correct epitopes than do the other methods, and it has significant improvement over MixMHCpred and MHCflurry (one-sided Mann–Whitney *P*-values 1.3e-05 and 8.5e-16, respectively); the improvements as compared to the NetMHCpan methods are not significant. (*D*) Comparison of MHCGlobe to other pan-MHC methods when predicting MHC–peptide binding for monoallelic cell lines (published with MHCflurry 2.0 O'Donnell 2020), as measured by area under the receiver operator curve (AUC). MHCGlobe performs significantly better than MHCflurry-2.0 BA, NetMHCpan-4.0 EL, and NetMHCpan-4.0 BA (one-sided Mann–Whitney *P*-values of 2.6e-3, 9.6e-4, and 3.7e-6, respectively). (*E*) For each MHC allele unseen during its training (10-fold cross validation, all data for an allele in one fold), MHCPerf’s output PPV (*x*-axis) as compared to the actual measured PPV of MHCGlobe for that allele when using data from the other nine folds (*y*-axis). The gray diagonal line indicates where measured PPV equals predicted PPV. (*F*) The Spearman’s rank and Pearson’s correlation coefficient (PCC) of MHCPerf’s predicted PPVs and MHCGlobe’s actual PPV (blue) as compared to the correlations obtained when predicting PPVs based on using the actual PPV of the test allele’s nearest training fold neighbor (gray).

Having shown that there are vast differences in the amount of binding data associated with each MHC allele, we next introduce a framework that both develops a pan-MHC model as well as uncovers how allele-level performance for diverse MHC alleles is affected by the availability of binding data. In particular, we devise two systems: (1) MHCGlobe, a state-of-the-art deep learning method for pan-MHC binding affinity (BA) prediction; and (2) MHCPerf, a method for estimating allele-level performance of MHCGlobe (Fig. 2). MHCGlobe takes as input a query MHC class I allele’s pseudosequence and a peptide sequence (8 to 15 amino acids in length) and outputs a score predicting the MHC–peptide BA value (Fig. 2*A*). MHCGlobe is an ensemble of neural networks trained on MHC–peptide binding data. MHCPerf (Fig. 2*B* and *SI Appendix*, Fig. S2) is a shallow neural network model that takes as input a query MHC allele’s pseudosequence as well as MHCGlobe’s training data to predict the Positive Predictive Value (PPV) MHCGlobe would achieve on that query MHC allele when trained on that dataset (*Materials and Methods*). For a query MHC allele, we measure PPV on a test dataset with a 99:1 ratio of nonbinding to binding peptides as the fraction of the top 1% of peptides scored by MHCGlobe that actually bind the query MHC allele; we use PPV to estimate per-allele performance as in most applications of MHC binding models, the top predictions are considered for downstream analysis and only actual binding pairs are of interest. In order to generate a training set for MHCPerf, for each MHC allele with enough binding data, we train MHCGlobe multiple times on different datasets (i.e., consisting of binding peptides for different sets of MHC alleles) and measure the actual PPV that MHCGlobe obtains for that MHC allele when trained on these different datasets (*Materials and Methods*). MHCPerf is trained on examples derived from this process, where each example consists of a query MHC allele, a target variable of the measured PPV of MHCGlobe on this allele when trained on a specific dataset, and features describing the relationship between the query MHC allele and this dataset (*SI Appendix*, Fig. S2 and Table S4). In principle, an approach such as MHCPerf can be used to predict the performance of any existing pan-MHC model, as long as it is computationally feasible to train the pan-MHC model many times in order to generate the training data for MHCPerf. Together, MHCGlobe and MHCPerf provide a level of transparency unavailable for existing MHC binding prediction methods and will allow researchers to anticipate when model performance may be a limitation for specific MHC alleles of interest, and when variation in performance across alleles may confound downstream bioinformatic analyses.

### MHCGlobe Has State-of-the-Art Performance, and MHCPerf Accurately Estimates Per-Allele Performance.

To ensure the usefulness of MHCGlobe and MHCPerf to the broad research community, we start by evaluating MHCGlobe’s performance against performance of the available alternative methods NetMHCpan-4.1 (11), NetMHCpan-4.0 (12), MixMHCPred-2.1 (14), MHCflurry-1.2 (15), and MHCflurry 2.0 (16). We evaluate MHCGlobe performance on two benchmarks published with NetMHCpan4.1 and MHCflurry 2.0, enabling comparison to alternative methods that were benchmarked previously; results shown here for other approaches are as reported in these previous benchmarks (*Materials and Methods*). We first assess how well each method performs in identifying 1,660 known T cell (CD8+) epitopes from full protein sequences. Here, for each documented CD8+ T Cell epitope, we use each of the methods to rank the predicted BA of the epitope among all other 8-14-mer peptides from the same source protein sequence. MHCGlobe performs as well as or better than other methods in assigning higher binding affinities to known epitopes as compared to other peptides within the sequences (Fig. 2*C*). Next, we consider how well each of the methods predict MHC-presented peptides for 100 monoallelic engineered cell lines. MHCGlobe outperforms all other BA prediction methods in classifying peptides that either bind or do not bind the tested alleles (Fig. 2*D*).

To evaluate our second model, MHCPerf, we use 10-fold cross-validation and test MHCPerf’s ability to predict PPV for HLA alleles that it has not seen in its training (Fig. 2*E*). Predicted PPV by MHCPerf is highly correlated with measured PPVs, with PCC of 0.58 and Spearman’s rank correlation coefficient (SCC) of 0.56. Nielsen et. al. (10) showed that the measured performance of an allele is positively correlated with that of its most sequence-similar MHC alleles with available training data. We use this observation to create a “nearest neighbor” approach which predicts the PPV of a query allele as the measured PPV of the most similar allele with training data. MHCPerf greatly outperforms the nearest neighbor approach (PCC of 0.58 versus 0.35 and SCC 0.56 versus 0.33 for MHCPerf versus Nearest Neighbor) (Fig. 2*F*). Thus, MHCPerf newly offers the ability to reliably estimate allele-level performance for previously uncharacterized HLA alleles (i.e., those with no experimentally measured binding data), and opens up opportunities to assess the quality of MHC–peptide predictions across diverse sets of individuals.

### MHCGlobe Mitigates Disparities Across Racial and Ethnic Populations but Inequities Remain.

**Fig. 3.**
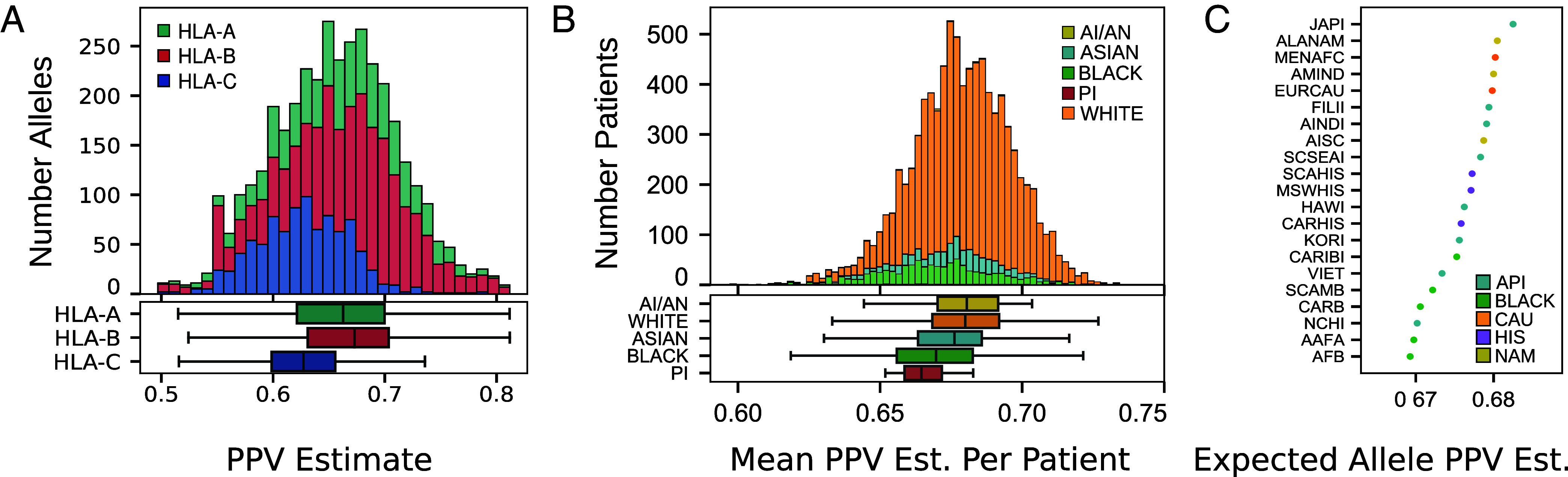
Assessment of MHCGlobe’s predicted performance per-allele, per-individual, and per-broad racial group. (*A*) For each of 3,804 distinct MHC pseudosequences, MHCPerf predicts the expected PPV that MHCGlobe will have on a test set of data for that allele. Shown is a histogram (*Top*) and boxplot (*Bottom*) of the predicted PPVs. Expected performance differs between HLA genes (Mann–Whitney two-sided *P*-values of 0.003, 2.3e-41, 6.7e-76 for comparisons between HLA-A and HLA-B, HLA-A and HLA-C, and HLA-B and HLA-C, respectively). 72 alleles with outlier values defined as ± 1.5 times the interquartile range (IQR) are not depicted here. (*B*) (*Top*) The number of individuals from TCGA with a given patient-level PPV score, which is computed as the average of the PPV estimates corresponding to each of their six class-I HLA alleles. Colors represent the race categorization recorded for each patient. (*Bottom*) The distribution of patient-level PPV scores aggregated by race. There are significant differences in the expected performances of MHCGlobe, when comparing White and Asian populations, White and Black populations, and White and Pacific Island populations (two-sided Mann–Whitney *P*-values of 1.56e-12, 3.63e-50, and 0.004, respectively); the expected performances of MHCGlobe are not significantly different when comparing White and AI/AN populations. (*C*) The expected PPV estimate for MHCGlobe, as estimated by MHCPerf, for an allele sampled from each of the 21 detailed racial and ethnic populations. Colors represent the broad race and ethnic categorization associated with each of the detailed race or ethnic categorizations as reported by Gragert et al. (2013), as in Fig. 1*D*.

We next use MHCPerf to quantitatively assess MHCGlobe’s predicted performance across alleles, individuals, and racial and ethnic groups. Remarkably, MHCGlobe has reasonably high estimated performance for the majority of alleles (median predicted PPV 0.65), which is notable considering that 87% of the MHC pseudosequences are not associated with any binding data. However, we observe large variation in MHCGlobe’s estimated performances, ranging from PPVs of 0.27 to 0.94, across the 3,804 distinct MHC pseudosequences (Fig. 3*A*). Consistent with the distribution of the known binding data, pseudosequences corresponding to HLA-B alleles overall have the highest predicted performance, followed by HLA-A and then HLA-C alleles. For each individual profiled in TCGA, we can compute the estimated performance of all of their MHC alleles by averaging the MHCPerf estimated PPVs across their six alleles. We see a clear spread in performance scores across individuals, ranging from 0.60 to 0.73 (Fig. 3*B*). Notably, MHCGlobe’s pan-MHC model partly mitigates the data disparity, with the differences between individuals with White and Asian ancestries and between individuals with White and Black ancestries not as statistically significant when comparing MHCGlobe’s expected performance as they are when comparing the amount of available data (compare with Fig. 1*C*). However, we still observe significant differences in the expected performances of MHCGlobe across populations, and this overall pattern is corroborated when computing the expected performance estimate for a random HLA allele selected from each of the 21 racial and ethnic groups assessed earlier for data coverage (Fig. 3*C*). Based on these assessments, the effect of data sharing in pan-MHC modeling is unlikely to completely mitigate existing data coverage inequalities across individuals, races, and ethnic groups—and indicates that efforts toward fair pan-MHC binding prediction methods and downstream technologies should prioritize strategic data collection to address these disparities.

### An Algorithmic Strategy Based on MHCPerf Identifies HLA Alleles for Data Collection in Order to Mitigate Performance Disparities Across Classical HLA Alleles.

**Fig. 4.**
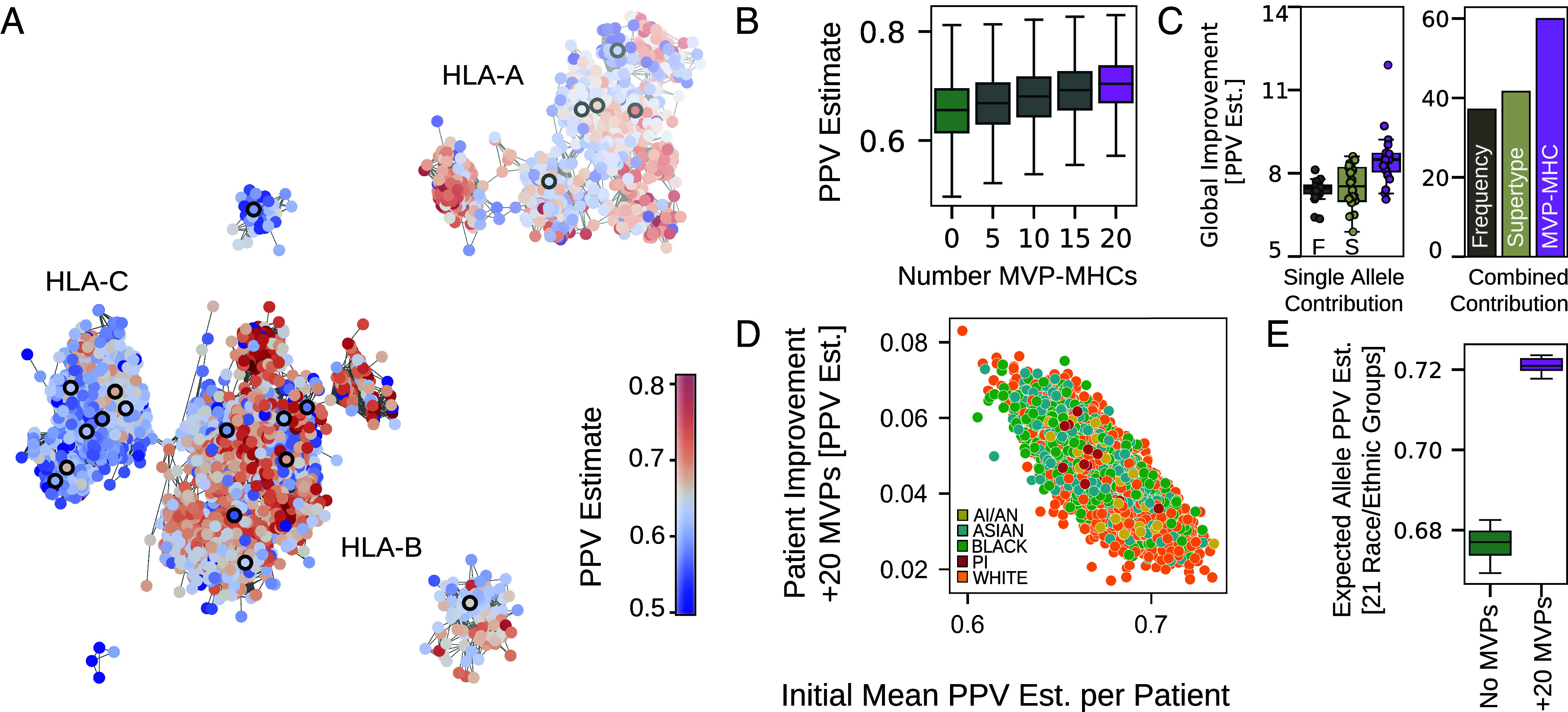
Toward fairness in pan-MHC modeling. (*A*) Network of distinct HLA pseudosequences and their relationships as in Fig. 1*E*, but colored by their estimated PPV. Nodes with a thick black border represent the pseudosequences of first 20 MVP-MHC alleles selected for data prioritization. Color scale is limited to ± 1.5 times the IQR to accentuate contrast among the majority of alleles, meaning outlier values are colored the same as those with values at the boundary of ± 1.5 * IQR. (*B*) Incremental addition of 4,000 data points for each of the top 5, 10, 15, or 20 MVP-MHCs, followed by retraining MHCGlobe, is expected to improve performance across HLAs. The purple boxplot on the right corresponds to adding data for all of the top 20 MVP-MHC alleles, and then retraining MHCGlobe. The green boxplot on the left corresponds to the scenario where no alleles are given additional data. (*C*) Comparison of three approaches to select MHC alleles to prioritize for data collection as assessed by predicted Global PPV Improvement for all distinct HLA pseudosequences. Global PPV Improvement is defined as the sum across all 3,804 distinct HLA pseudosequences of the difference between PPV estimates after versus before adding 4,000 hypothetical data points to the specified alleles. The three approaches to allele selection are Frequency (Dark Brown); Supertype (Gray), and MVP-MHC (Purple). (*Left*) Global improvement in PPV estimates with the hypothetical data given to a single selected MHC allele. For each of the three approaches for allele selection, boxplots give the predicted Global PPV Improvement when adding data for each one of the 20 selected alleles individually. The MVP-MHC approach leads to better predicted Global PPV Improvement (Mann–Whitney one-sided *p*-values of 4.16e-5 and 4.6e-4 comparing MVP-MHC to Frequency or Supertype, respectively). (*Right*) Predicted Global PPV Improvement if data were added for all 20 selected alleles by each corresponding approach. (*D*) Improvement in individual-level PPV score (computed as the change in average estimated PPV across their six class-I HLA alleles) for individuals profiled in TCGA after adding 4,000 data points to all of the top 20 MVP-MHC alleles plotted against the current individual-level PPV estimate without additional data for MVP-MHC alleles. Colors correspond to patient race (as in Fig. 1*C*). Patients with lower initial PPV estimates (*x*-axis) tend to have larger improvements (*y*-axis) in their patient-level PPV score. (*E*) Comparison of expected allele PPV estimates for the 21 race and ethnic groups (from Fig. 1*D*) when 4,000 data points are added to the top 20 MVP-MHC alleles or not. Across race and ethnic groups, adding data for the MVP-MHC alleles increases expected performance for MHCGlobe (*P*-value of 3.3e-10, one-sided Mann–Whitney test) as well as reduces its variation.

To provide steps forward, we next utilize the capabilities of MHCPerf to (1) identify alleles with poor estimated performance in pan-MHC modeling and (2) identify key MHC alleles—dubbed MVP-MHCs (“Most Valuable Player” MHCs)—to prioritize for data collection in order to strategically improve performance broadly across numerous alleles and reduce inequities in pan-MHC models. In this section, all mentions of performance refer to estimated performance by MHCPerf. Visualizing the network of HLA pseudosequences colored by allele-level performance, rather than data, we observe distinguishable regions of the network with either strong or relatively weaker performance (Fig. 4*A*). Regions of poor performance define blind spots of MHCGlobe in MHC sequence space which could be strategically addressed with collection of binding data for alleles in those regions.

We therefore develop a greedy approach that leverages MHCPerf to tackle the problem of identifying MVP-MHC alleles, which if additional data were collected for, would have the most “beneficial” impact in improving MHCGlobe “weak spots” in performance. The greedy algorithm iteratively selects the MVP-MHC alleles that best improve the performances for alleles where MHCGlobe is underperforming (*Materials and Methods*). We use our algorithm to select the top 20 MVP-MHC alleles to prioritize for strategic data collection to demonstrate the advantage of our approach. Incremental addition of data for these selected 20 MVP-MHC alleles raises the distribution of PPV estimates across alleles (Fig. 4*B*). Moreover, the spread in the estimated performances across alleles declines, with the SD decreasing from 0.062 initially to 0.053 after data for 20 alleles is added.

We highlight the putative advantage of selecting MVP-MHC alleles chosen by our algorithm compared to two alternative strategies, population frequency-based or the supertype-based prioritization of alleles. The population frequency-based approach chooses the 20 most common HLA alleles for target data collection [as estimated across 497 global population samples (22)]. The supertype-based approach selects 20 alleles for prioritization by choosing one allele from each of 20 HLA “supertypes” associated with the least amount MHC binding data; MHC alleles are all classified into supertype clusters based on their sequence similarity (23). To compare each data collection strategy, we use MHCPerf to estimate how much performance would be improved globally (across the 3,804 HLA pseudosequences) if 4,000 additional binding records were collected per allele selected by these approaches (Fig. 4*C*). The global improvement across the pseudosequences, computed as the difference in the sum of PPVs across alleles, is greater for MVP-MHC alleles, considered individually, as compared to alleles selected by either of the two alternative methods (Fig. 4 *C*, *Left* panel). This improvement is amplified considering the impact of adding data for all 20 alleles together (Fig. 4 *C*, *Right* panel). Remarkably, prioritizing the top 20 MVP-MHC alleles is expected to improve patient-level performance for all of the TCGA patients, but yields the greatest improvement for patients currently with poorest performance (Fig. 4*D*). We next assess the impact of adding data for the MVP-MHC alleles on estimated performance across racial groups and find that the expected performance of MHCGlobe across the 21 racial and ethnic groups (*Materials and Methods*) is higher with additional data for the MVP-MHCs (Fig. 4*E*). Moreover, the variability narrows across groups, which suggests that obtaining data for these prioritized MVP-MHCs should yield more equitable predictions of MHC binding across racial groups.

## Discussion

Computational methods for predicting MHC binding play a critical role in personalized cancer immunotherapies and effective vaccine design. In order to ensure that cutting-edge treatments benefit individuals from diverse racial and ethnic groups, it is essential that MHC binding models perform equitably across populations. Here, we demonstrate that peptide–MHC binding data is highly imbalanced across the human HLA alleles, and this results in disparate data coverage across individuals and across racial and ethnic populations. To assess the impact of this data imbalance, we paired the development of a pan-MHC method, MHCGlobe, for predicting MHC–peptide binding with an approach, MHCPerf, that estimates MHCGlobe performance for all MHC alleles, including those with no binding data. We demonstrate state-of-the-art performance for MHCGlobe and show that MHCPerf is able to reliably predict allele-level MHCGlobe performance values for alleles unseen in MHCGlobe’s training, only requiring the pseudosequence for the query MHC allele along with the training data used to train MHCGlobe.

While MHCPerf uncovers that MHCGlobe has high estimated performance for the majority of alleles—even for those that are not associated with any binding data—it also finds considerable variation in MHCGlobe’s performances across alleles, which leads to inequity in the quality of MHC–peptide binding predictions across racial and ethnic groups. Since MHCGlobe is trained on similar data as previous approaches, we expect that all widely used MHC class I predictors available today are likely to perform better as a whole for individuals with White ancestry than they do for individuals with Black or Asian ancestry. Indeed, in agreement with this, we find that MHCGlobe’s performance on different alleles in the FRANK dataset is highly correlated with that of NetMHCPan-4.1 (SCC of 0.94). In theory, a framework like MHCPerf can—and should—be utilized to estimate the per-allele performance of any machine learning method for MHC–peptide binding, as long as it is computationally feasible to train the method on numerous subsets of the training data and measure performance.

To mitigate inequity in pan-MHC model performance, we propose a heuristic strategy for targeted data collection; our analysis suggests that such a strategy would lead to more equitable predictions by improving predictions more for underperforming alleles. In the future, it may be fruitful to define specific objective functions (e.g., to improve performance for specific sets of alleles for which predictions are particularly poor, or to improve performance for certain populations) and develop general optimization approaches to find solutions. Overall, identifying MHC alleles for targeted data collection is an important approach for obtaining equitable MHC–peptide predictions.

Another parallel avenue to obtain more equitable MHC–peptide binding predictions is to try to modify the training of neural networks. Here, all training data contributed equally to the loss function used for training. It may be beneficial to modify training so as to upsample or upweight data for less well-studied MHC alleles (24, 25). However, since alleles also have varying amounts of sequence similarity to each other, and sequence-similar alleles tend to have relatively similar binding profiles, it is likely that more specialized schemes that consider the similarity of alleles to each other will be necessary. Alternatively, the loss function used here minimizes error across all data points; the use of other loss functions that prioritize different goals may also be beneficial for obtaining more equitable predictions (26). For any modification in training, it will be necessary to first verify that the overall performance of the model is state-of-the-art and then estimate (using an approach such as MHCPerf) allele-level and population-level performance in order to characterize whether MHC predictions are equitable.

In conclusion, our work has highlighted the importance of intentionally developing pan-MHC models that not only have state-of-the-art performance but also work well across diverse sets of alleles. We have presented an initial approach to estimate per-allele performance and have shown how it can be used to assess equity in MHC binding predictions and guide targeted data collection. The approaches introduced here lay the groundwork for further development of equitable MHC binding models, a key step in ensuring that future advances in personalized immunotherapies benefit genetically diverse individuals.

## Materials and Methods

### Peptide–MHC Binding Data.

Our dataset is derived from the Immune Epitope Database (IEDB) (https://iedb.org/downloader.php?file_name=doc/mhc_ligand_full_single_file.zip) (27) and the MHCflurry 2.0 dataset (16). Our dataset consists of class I MHC alleles, with a linear peptide epitope and peptide lengths between 8 and 15 amino acids. MHC–peptide binding data is either associated with a quantitative BA, or is qualitative (binding or not) and comes from elution experiments (EL) from either multiallelic (MA) or single-allelic (SA) cell lines. Preprocessing of the data and assignment of peptides from MA data to specific MHC alleles is described in detail in *SI Appendix*. We prune the data so that each distinct MHC–peptide appears only once, where the mean BA measurement is used when multiple binding affinities are recorded for the same MHC–peptide pairing. After preprocessing steps, we have 43,626 BA, 378,039 MA, and 518,573 SA positive MHC–peptide examples (full description in *SI Appendix*, Table S2). All positive qualitative records are assigned a quantitative affinity value of 100 nM and measurement inequality of “<”, which indicates that positive examples should be assigned a value less than 100 nM by the neural network (24).

Synthetic negatives are generated by sampling *k*-mer amino acid subsequences uniformly at random from protein sequences in UniProt (downloaded 10/2020). For each allele, synthetic negatives are added at five times the total number of positive data points for that allele, with an equal number of different lengths (8 to 15-mers) represented in the sampled synthetic negatives. Synthetic negatives are assigned a quantitative value between 20,000 to 50,000 nM, sampled uniformly, and measurement inequality of “>”.

MHC proteins are represented as pseudosequences, using the 34 amino acids in positions 31, 33, 48, 69, 83, 86, 87, 90, 91, 93, 94, 97, 98, 100, 101, 104, 105, 108, 119, 121, 123, 138, 140, 142, 167, 171, 174, 176, 180, 182, 183, 187, 191, and 195, as previously described (11, 16). MHC pseudosequences containing an unknown residue, “X”, in their 34-length pseudosequence are excluded.

### Construction of HLA Pseudosequence Network.

The network consists of 3,804 nodes representing distinct class I HLA allele pseudosequences (HLA-A, -B, -C only). Pseudosequences used in the current study were released with MHCflurry 2.0 (16). Edges between nodes correspond to similar pseudosequences defined as having a BLOSUM62 distance within the range 0-0.1. The BLOSUM62 distance of two MHC pseudosequences A and B is defined as in ref. 14 as $d=1-\frac{s\left(A,B\right)}{\sqrt{s\left(A,A\right)\cdot s\left(B,B\right)}}$ where $s\left(A,B\right)$ is the BLOSUM62 alignment score (28) of the pseudosequences. Omitted from the visualization are 11 of the 3,804 (0.3%) pseudosequences that are not similar enough to any other in our dataset to have a connecting edge in the network.

### Determining HLA Haplotypes for TCGA Data.

Whole Exome Sequencing (WXS) data was downloaded for all normal (noncancer) TCGA samples from the GDC Browser (https://portal.gdc.cancer.gov), and Optitype (29) was used to determine HLA haplotypes. A preliminary processing step to Optitype is to use RazerS3 (30) to filter WXS reads to those able to align to the MHC allele reference library. Optitype was then run on each sample with default parameters. TCGA individuals with discrepancies across any of the six classical class I HLA alleles across multiple associated samples were excluded from this study. This resulted in high confidence HLA haplotypes for 8,942 individuals. In our dataset, we observe 334, 163, 68, 34, and 145 distinct alleles for individuals categorized by TCGA as White, Black, American Indian or NAM, Native Hawaiian or other Pacific Islander, or Asian, respectively.

### MHCGlobe.

#### Overview of MHCGlobe.

MHCGlobe is an ensemble of neural networks, whose design and model selection is described below (see Ensemble Model Selection). Each neural network outputs a value between 0 and 1, corresponding to a log transformed BA value (“nM” units), where measured affinity value, *a*, is transformed by 1-log(*a*)/log(50,000) as introduced by Nielson et al. (31). For training, we utilize a modified mean squared error loss that incorporates inequalities associated with each peptide–MHC pair so as to avoid penalizing predictions which fall within a specified range for BA labels associated with semiquantitative and qualitative binding records (16). Each MHCGlobe neural network was trained using RMSprop gradient descent optimization within the TensorFlow (https://www.tensorflow.org/) training functions. In order to utilize BA and elution data for peptide–MHC binding, a modified mean squared error loss function that handles measurement inequalities (“<”, “=”, “>”), introduced by MHCflurry (15, 16), was used.

#### Peptide and MHC input representations.

All peptides are represented as 15-mers in the manner introduced by MHCflurry 1.2 (15). For peptides shorter than 15 residues, the first and last four residues are placed at the respective ends of the 15-mer and the central residues of the peptide are added to the center of the 15-mer representation. Unfilled positions in the 15-mer are set to an “X” residue. The representations of amino acids were treated as a hyperparameter, where each amino acid is represented either by a one-hot encoding (a vector of 20 zeros with a 1 in the position representing the amino acid), or by the corresponding row from BLOSUM62 which represents the similarity of that amino acid to all other amino acids. The unknown residue X is always encoded as a vector of 20 zeros.

#### Overview of strategy for choosing MHCGlobe model architecture.

Our goal was to optimize MHCGlobe’s architecture without any exposure to the classical HLA genes, HLA-A, HLA-B, and HLA-C, which were reserved for the Leave-N-Out (LNO) cross validation (described in following sections, and which were used to measure how allele-level performance changes as a function of the binding data made available to MHCGlobe for training). Optimal hyperparameters were selected for the MHCGlobe ensemble by identifying neural networks trained on nonhuman MHC data that could best predict binding data for nonclassical class I MHC alleles from human genes (i.e., HLA-E and HLA-G) (*SI Appendix*, Fig. S1). MHCGlobe’s architecture was thus fixed prior to exposure to any data from classical HLA alleles. In addition to having classical HLA data available for LNO cross validation, we reasoned that hyperparameters enabling a pan-MHC model to predict peptide–MHC binding for MHC alleles from unseen species and on nonclassical class I MHC alleles would encourage the selection of hyperparameters that yield more generalizable models and thus are more desirable for a pan-MHC method.

#### Hyperparameter optimization.

Hyperparameter optimization was performed using Optuna (32). Optuna optimizes hyperparameters by monitoring and minimizing mean squared error on a held-out dataset, referred to here as the *optuna_set*. The space of hyperparameters is explored within an Optuna study, wherein Optuna iteratively samples and tests new hyperparameter combinations with sampling guided by performance measurements on the optuna_set from previous iterations. We used 100 iterations per Optuna study, and 15 Optuna studies in total. In total, this yielded 1,500 explored hyperparameter settings from which we eventually chose individual deep neural network models to combine into an ensemble predictor. The hyperparameters we considered define neural network architectures and regularization parameters, and include the gradient descent algorithm’s parameters (*SI Appendix*, Table S3).

For each Optuna study, the nonhuman MHC data were newly split into a training, validation, and optuna_set at a 4:1:1 ratio. The validation fold is used for early stopping when training the neural networks. Splits were created using distinct peptide–MHC pairs to avoid overlap between any two splits. Peptide–MHC pairs which are repeatedly observed in the data were always in the same data split. In order to ensure Optuna sufficiently tested neural networks of varying depths, we did not allow Optuna to sample the number of hidden layers as a hyperparameter for MHCGlobe; instead, the number of hidden layers was fixed to either 1, 2, or 3 layers and each tested for five of the 15 studies. All Optuna studies utilized a different splitting of nonhuman MHC binding data into training, validation, and optuna_set.

#### Ensemble model selection.

To compose the ensemble, we iterated through each of the trained 1,500 models and added the model to the ensemble which best improved ensemble performance on the nonclassical human HLA data and repeated until no further improvement was achieved. The predicted binding score for an ensemble is taken as the arithmetic mean of each of the neural network predictions within the ensemble. Following this approach, we obtained an ensemble composed of three neural networks. Two of the selected neural networks have three fully connected hidden layers, and the third has two fully connected hidden layers. All three selected neural networks utilize at least one skip connection which allows information to flow through a given hidden layer and bypass the same hidden layer before being concatenated and passed to the following model layer. Since initialization values for weights and bias parameters of neural networks are important for learning and performance (33), the initialization weights and biases for these three networks are set to be the same as they were prior to training on nonhuman MHC data and the same initialization weights and biases are used any time the particular neural network model is retrained. Once the ensemble model was selected, the full MHCGlobe model was trained on all available MHC binding data.

#### Early stopping for MHCGlobe training.

For all MHCGlobe variants trained in this study, early stopping was used to prevent overfitting. MHCGlobe was trained for a maximum of 200 epochs, and halted by early stopping if performance on the validation set did not improve over 20 epochs by at least 0.0001. An allele-balanced training and validation set was created by partitioning 1/5th of the binding data for each allele to the validation set. As certain peptide–MHC records were observed multiple times in the available MHC binding data, training and validation partitions were performed using distinct peptide–MHC records, and repeated observations were added back into their respective partitions following partitioning.

#### Benchmarking MHCGlobe performance.

We benchmarked MHCGlobe using datasets published with NetMHCpan 4.1 (11) and MHCflurry 2.0 (16). In all benchmark comparisons to other methods, the MHCGlobe model was retrained after removing all peptide–MHC pairs that occur in the benchmark test set of interest. The NetMHCpan 4.1 benchmark (11) consists of 1,660 protein sequences with documented CD8+ T-cell epitopes. For each documented CD8+ T Cell epitope, the epitope should have a higher BA than other 8 to 10-mers peptides from the same source protein sequence. For each sequence, predicted binding scores of all peptides within it are rank normalized to obtain a FRANK score for each documented T cell epitope, where peptides with the highest predicted BA are given a score of 0 and the peptides with the lowest predicted BA are given a score of 1. Lower FRANK scores for the correct epitopes correspond to better performance. To make Fig. 2*A*, FRANK scores for NetMHCpan 4.1, NetMHCpan 4.0, MixMHCpred 2.1, and MHCflurry 1.2 were taken from *SI Appendix*, Table S7 of paper (11), and the FRANK scores for MHCGlobe were computed in this study using MHCGlobe binding predictions on the same benchmark. The MHCflurry 2.0 benchmark (16) consists of 100 monoallelic cell lines where peptides that either bind or do not bind the HLA allele are given (34). For each sample, an area under the receiver operator curve (AUC) is computed to measure performance. To make Fig. 2*B*, binding prediction scores for MHCflurry 2.0, NetMHCpan 4.0 BA, and EL are given in Supplemental Table S2 of O’Donnell et al. (16) and MHCGlobe binding scores were added to compute the AUC for MHCGlobe. While MixMHCPred was originally included in the benchmark of MHCflurry 2.0 for a subset of MHC alleles, we exclude MixMHCPred here as we consider only pan-MHC methods that are able to make predictions for all class I MHC alleles in the benchmark.

#### Summary of MHCGlobe design.

We briefly summarize the methodological similarities and differences of MHCGlobe as compared to the widely used methods that we compare to [the NetMHCPan methods (11, 12), MHCflurry (15, 16), and MixMHCPred (14)]. All methods compared are trained on class I MHC binding data from the IEDB. NetMHCpan is additionally trained on in-house datasets which were not made public. MHCGlobe, NetMHCpan, and MHCflurry all consist of an ensemble of neural networks (1 to 3 layers, and 3 to 40 networks). MHCGlobe utilizes the same sequence padding strategy as MHCflurry (15) to handle variable length sequences. MixMHCPred utilizes an algorithm based on mixture models, and thus is not based on neural networks as the other described methods are. Perhaps the most significant change of the MHCGlobe method as compared to previous approaches is in hyperparameter selection (*SI Appendix*, Fig. S1), where MHCGlobe’s hyperparameters are chosen based on nonhuman and nonclassical HLA alleles, allowing the same hyperparameters to be used to train multiple version of MHCGlobe for the generation of training data for MHCPerf.

### Creating the MHCPerf Training Dataset via LNO Cross Validation.

#### Choosing MHCGlobe training data subsets.

A schematic of the LNO cross validation is given in *SI Appendix*, Fig. S2. MHC alleles that had at least 50 distinct EL binding records (derived from either SA or MA data) were considered to have sufficient data to evaluate allele-level performance and were selected as test alleles. This yielded 125 test alleles. We generated 10 distinct MHCGlobe training sets for each test allele, where the first training set excluded data from the test allele. Successive training sets excluded all binding data from the next most similar allele to the test allele (using the BLOSUM62 distance defined above), such that at least 10 additional binding records had to be excluded; if fewer than 10 binding records were excluded, then an additional neighboring allele is excluded from the particular MHCGlobe training set. In total, 1,250 MHCGlobe training datasets were created.

#### Training LNO versions of MHCGlobe.

A distinct version of MHCGlobe was trained for each of the 1,250 LNO training sets, following the same training protocol as the full model, including always being initialized with the same weights and bias values prior to being trained on binding data. The 1,250 models were trained in parallel using g3.4xlarge EC2 instances from Amazon Web Services (AWS).

#### Measuring allele-level performance of MHCGlobe.

For each of the 125 test alleles, MHCGlobe’s PPV for that allele was measured for each of the 10 associated LNO MHCGlobe models. Each of the 125 test sets is composed of positive elution records of the corresponding test allele with the addition of synthetic negative peptides sampled at 99:1 with peptides of lengths 8-10 amino acids equally represented. PPV is computed as the proportion of positive labeled peptides in the top 1% of BA predictions made by the corresponding LNO version of MHCGlobe.

### Modeling Allele-Level PPV via MHCPerf.

#### Featurization.

The relationship between a query MHC allele and the binding data used to train MHCGlobe is used to create 63 features for MHCPerf that can be conceptually grouped into five categories (*SI Appendix*, Fig. S2 and Table S4). The first quantifies the BLOSUM62 sequence distance between the query MHC and each of the 10 most similar alleles with binding data included in the MHCGlobe training set. The second feature set quantifies the number of positive binding data points each of those 10 most similar alleles has in the training dataset. The third feature set quantifies the amount of positive binding data corresponding to training set alleles in one of eight sequence distance bins (0 to 0.1, 0.1 to 0.2, 0.2 to 0.3, 0.3 to 0.4, 04 to 0.5, 0.5 to 0.6, 0.6 to 0.7, 0.7+) representing the sequence distance between the query allele and the training set alleles. The fourth feature set quantifies the number of positive training data points corresponding to each of the 34 residue-position pairs of the query MHC pseudosequence. The fifth feature set is a single value quantifying the total number of positive binding records in the training dataset. After MHCPerf was trained, we computed the SCC of each feature to MHCPerf’s estimated performance of MHCGlobe, and found that the features that measure the distance of the test allele to the nearest training alleles are most important for MHCPerf’s prediction.

#### MHCPerf PPV-balanced folds.

MHCPerf training data were split into PPV-balanced folds during the hyperparameter selection procedure and during 10-fold cross validation for MHCPerf performance evaluation. That is, because it is rare that MHCGlobe’s performance was either high or very low, in order to make each fold more representative of the full MHCPerf training set, we attempted to balance measured PPV across fold splits while keeping all instances corresponding to a given test MHC within the same fold. To do this, for each test allele, the mean PPV for MHCGlobe was first computed across the ten instances of LNO measurements. Then, test alleles were sorted by their mean LNO PPV and iteratively assigned to one of the folds.

#### MHCPerf architecture.

MHCPerf is a single shallow neural network, consisting of a single hidden layer that contains ten units with ReLU activation and a dropout rate of 0.25, followed by a single output node with sigmoid activation.

#### MHCPerf trainings.

MHCPerf was trained with RMSProp (Root Mean Squared Propagation) for 1,000 epochs. Minibatch training was employed with 10 records per batch. Early stopping was used and halted training if performance on the validation set did not improve over 100 epochs. For performance benchmarking, training and validation folds were generated from the full training set utilizing the KFold function from scikit-learn with parameters, *n _ splits=10* and *shuffle=True*. In this scenario, the model is trained on nine of the 10 data partitions and the 10th partition is held out of training to test model performance. This is repeated until each fold has served as the test set. The final MHCPerf model is retrained on all data.

### MVP-MHC Algorithm.

The goal of the MVP-MHC algorithm is to identify key MHC alleles, for which collecting additional data would increase the expected performance of MHCGlobe on alleles for which it is currently underperforming. As MHCPerf and MHCGlobe make predictions on pseudosequences, the MVP-MHC algorithm is run on pseudosequences; for simplicity, we refer to each pseudosequence as an allele and assume that data would be collected for a single allele for a pseudosequence. The MVP-MHC (Most Valuable Player-MHC) algorithm utilizes MHCPerf to compute, for every allele, the effect of adding data for that allele on the performance of every other classical HLA allele. For each allele *j,* we compute an “impact” vector *v_j_* of dimensionality equal to the number of alleles, where the *i*-th element of the vector is equal to 0 if the estimated PPV of *i*-th allele is greater than the median estimated PPV across all alleles, and is otherwise set equal to the minimum of (1) the difference between the predicted PPV for allele *i* after adding 4,000 data points to allele *j* and the predicted PPV for allele *i* with the current data and (2) the difference between the median PPV for all alleles and the predicted PPV for allele *i* with the current data. In other words, this value is set to the amount of improvement for allele *i* when adding data for allele *j*, capped by how far the current performance for allele *i* is from the median performance. The total impact score for allele *j* is the sum of the elements of its impact vector. Intuitively, the impact vector for allele *j* measures how much closer the expected performance for each “underperforming” allele gets to the median PPV with the addition of data for allele *j*; alleles that already have performance above the median performance are not considered when selecting an MVP. The MVP-MHC algorithm greedily picks MVP alleles. That is, at each iteration, the allele *m* with highest impact score is chosen as an MVP. Then for every other allele *j*, in order to choose MVP alleles that are complementary to each other, its impact vector is updated to be *v_j_* - *v_m_*, with any negative entry of *v_j_* subsequently set equal to 0. In the next iteration, these updated impact vectors are used to choose the next MVP-MHC. This MVP-MHC selection process is repeated until the desired number of MVP-MHC alleles have been selected or until all MVP-MHC candidates have been selected.

### Data or PPV Estimate Expected Values for 21 Racial and Ethnic Groups.

The expected data coverage (respectively PPV estimate) for the 21 detailed racial and ethnic groups shown in Figs. 1*D* and 3*C* is computed as $x_{g}=\sum_{m}c_{m}\cdot f_{g,m}$, where $x_{g}$ is the expected value of the amount of data (respectively PPV estimate) for a given racial or ethnic group *g*, $c_{m}$ is the amount of data (respectively PPV estimate) for allele *m*, and $f_{g,m}$ is the frequency of allele *m* in the given group [obtained from Gragert et al. (19)]. Allele frequencies within each group were normalized so that alleles for each of the associated HLA-A, HLA-B, and HLA-C genes together sum to one. Thus, the expected value represents the amount of data or PPV estimation for the average allele sampled from a given racial or ethnic population.

## Supplementary Material

Appendix 01 (PDF)

## Data Availability

Software data have been deposited in https://github.com/ejglynn/mhcglobe (35). Previously published data were used for this work (IEDB Dataset) (27).
