# Supplementary material for: Toward equitable major histocompatibility complex binding predictions
Source: Proc Natl Acad Sci U S A. 2025 Feb 18;122(8):e2405106122. doi: 10.1073/pnas.2405106122 (PMC11874272; doi:10.1073/pnas.2405106122)
Supplement: Supplementary file 1 — Appendix 01 (PDF) [file pnas.2405106122.sapp.pdf]

Supplement

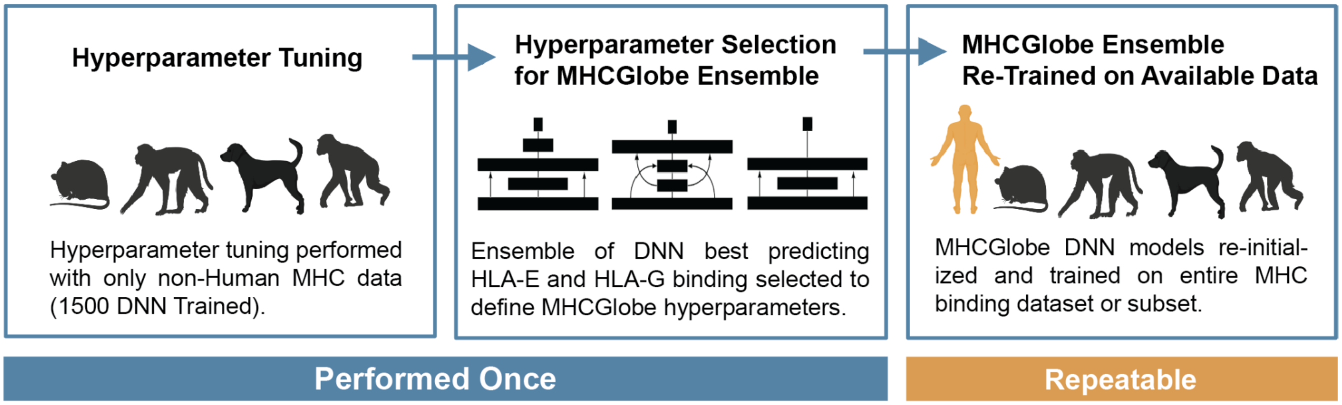

Supplementary Figure 1. Schematic of novel strategy for pan-MHC model hyperparameter selection, ensemble construction, and training used for MHCGlobe. In order to enable the Leave-N-Out cross validation with MHCGlobe on classical class I HLA data, MHCGlobe hyperparameters were first optimized utilizing non-Human MHC binding data where 1500 distinct Deep Neural Networks (DNN) were trained. To assemble the best subset of DNN for the MHCGlobe ensemble model, DNN trained on non-Human data were added to the MHCGlobe ensemble based on performance of HLA-E and HLA-G binding data (non-classical HLA alleles). The DNNs selected for the MHCGlobe ensemble were fixed, enabling MHCGlobe to be re-trained with any subset of classical class I HLA alleles in the Leave-N-Out cross validation. Figure created with bioRender.

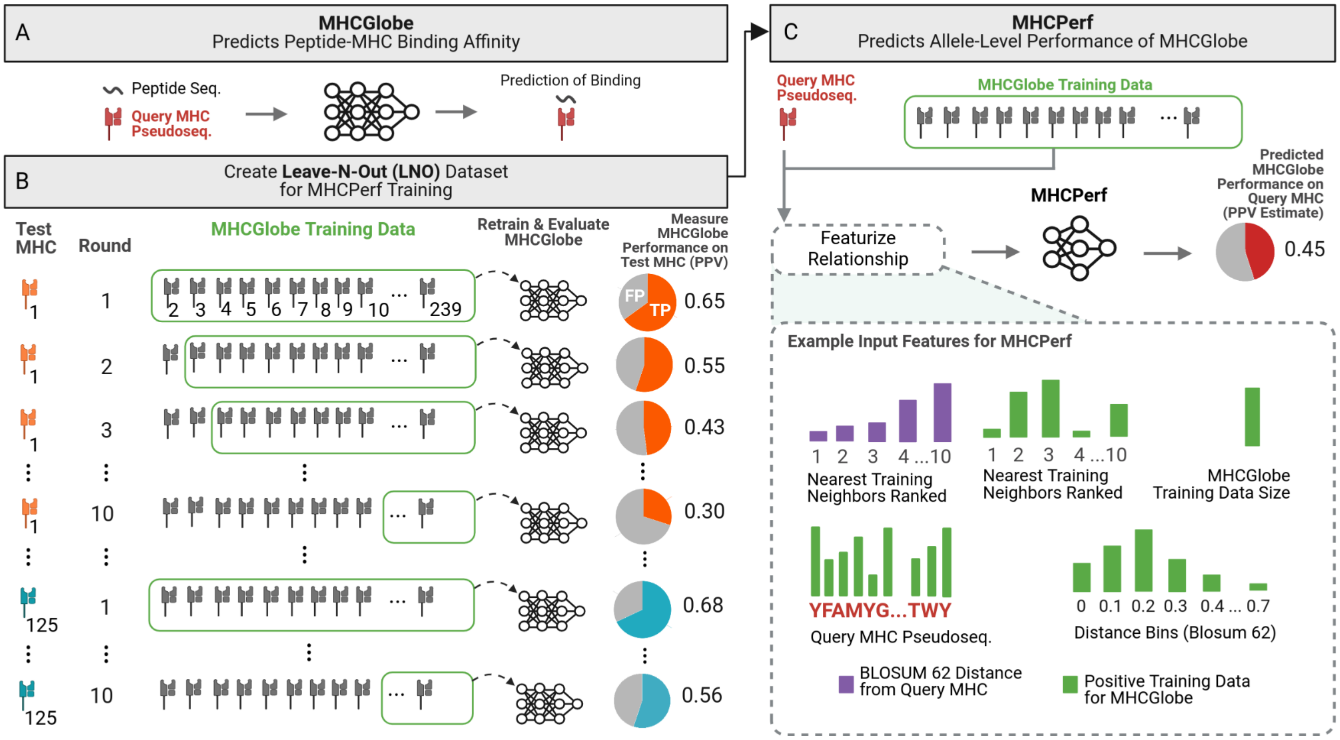

Supplementary Figure 2. Schematic of new methods, creation of MHCPerf training dataset, and MHCPerf input features. (**A**) As in Figure 2, MHCGlobe takes as input a query class I MHC allele's pseudosequence and a peptide sequence (8-15 amino acids in length) and outputs a score predicting the MHC-peptide binding affinity value. MHCGlobe is an ensemble of neural networks trained on MHC-peptide binding data. (**B**) Leave-N-Out cross validation to build a training dataset for MHCPerf. Each of 125 HLA alleles with sufficient data serves as a test MHC dataset for 10 rounds of cross-validation testing. In each round, the training set is altered such that MHCGlobe is trained on successively dissimilar MHC data by exclusion of neighboring alleles from the training dataset. For each of the 10 rounds, the actual performance of MHCGlobe on data for the test MHC is recorded (last column with pie chart and performance). (**C**) As in Figure 2, MHCPerf takes as input a query MHC allele's pseudosequence and uses MHCGlobe's training data to predict MHCGlobe's measured Positive Predictive Value (PPV) for that allele when trained on that data; this indicates the expected performance MHCGlobe would achieve on a test binding dataset for the query MHC allele (See Methods). The 1250 examples are generated from the data collected by the Leave-N-Out cross validation and consist of a test allele, features describing the relationship between the test allele and the data MHCGlobe is trained on, and the measured PPV of MHCGlobe on the test allele when trained on that dataset. In the bottom right, we give a schematic representation of MHCPerf's feature sets used to describe the relationship between a query MHC pseudosequence and the MHCGlobe training data (see Supplemental Table 4). Figure created with bioRender.

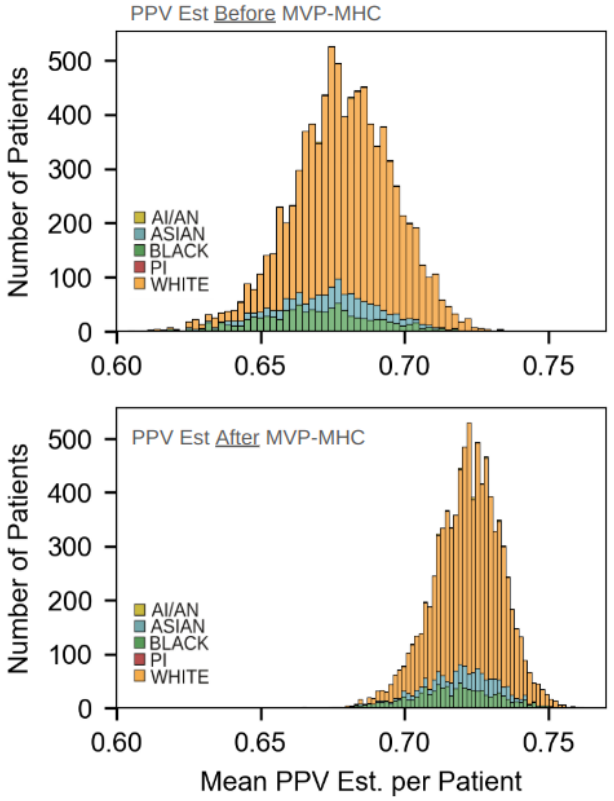

Supplementary Figure 3. The estimated performance of the six MHC alleles held by TCGA patients before (top) and after (bottom) the additional 4,000 hypothetical MHC-peptide data points for each of the 20 MVP-MHC alleles selected by the MVP-MHC algorithm. The top selected MVP-MHC alleles in order from greatest impact are as follows: HLA-C*07:14, HLA-B*40:237, HLA-A*31:115, HLA-C*16:53, HLA-B*13:19, HLA-A*02:310, HLA-A*30:157, HLA-B*37:51, HLA-C*02:62, HLA-C*01:64, HLA-B*15:05, HLA-C*06:28, HLA-A*11:199, HLA-B*08:60, HLA-B*57:107, HLA-A*31:104, HLA-B*27:29, HLA-C*08:04, HLA-B*56:54, HLA-C*07:10.

**Race code**	**Detailed race/ethnic description**	**Broad race group**
AAFA	African American	BLACK
AFB	African	BLACK
AINDI	South Asian Indian	API
AISC	American Indian – South or Central Am.	NAM
ALANAM	Alaska native or Aleut	NAM
AMIND	North American Indian	NAM
CARB	Caribbean black	BLACK
CARHIS	Caribbean hispanic	HIS
CARIBI	Caribbean Indian	NAM
EURCAU	European caucasian	CAU
FILII	Filipino	API
HAWI	Hawaiian or other Pacific Islander	API
JAPI	Japanese	API
KORI	Korean	API
MENAFC	Middle Eastern or N. Coast of Africa	CAU
MSWHIS	Mexican or Chicano	HIS
NCHI	Chinese	API
SCAHIS	Hispanic – South or Central American	HIS
SCAMB	Black – South or Central American	BLACK
SCSEAI	Southeast Asian	API
VIET	Vietnamese	API

Supplementary Table 1. Labeling for 21 detailed racial and ethnic groups and corresponding to broad racial and ethnic groups for the allele frequency estimation published by Gragert *et. al.*[19].

	Positive	Negative
BA	43,626	110,541
SA	518,573	32,508
MA	378,039	36,340

Supplementary Table 2. Number of Positive and Negative peptide-MHC records for Binding Affinity (BA), Single Allelic (SA) Elution, and Multi-Allelic (MA) Elution data.

	**Neural Network 1**	**Neural Network 2**	**Neural Network 3**
**sequence_encoding**	ONE_HOT	ONE_HOT	ONE_HOT
**L1_reg**	1	0	0
**dense_units_1**	128	256	256
**dense_units_2**	128	512	512
**dense_units_3**	512	128	-
**skip_connection_1**	TRUE	TRUE	TRUE
**skip_connection_2**	TRUE	FALSE	NA
**epsilon**	6.85E-07	5.52E-07	3.17E-07
**rms_learning_rate**	0.00113393	0.00103829	0.00191475

Supplementary Table 3: MHCGlobe hyperparameters for each neural network in the ensemble model. Parameters listed from top to bottom correspond to: sequence encoding strategy for the peptide and the MHC pseudosequence; L1 regularization lambda value; the number of nodes for hidden layers 1, 2, and 3; boolean to use skip connection to add bypass connection over hidden layer 1 and/or hidden layer 2; epsilon and learning rate parameters for Root Mean Squared Propagation (RMSprop) gradient descent optimizer.

Feature Set	Number of Features	Description
Distance of 10 nearest training neighbors	10	The BLOSUM62 distance between the query MHC allele's pseudosequence and that for each of the 10 most similar MHC alleles with data in the MHCGlobe training set. Ordered by distance to query MHC.
Data of 10 nearest training neighbors	10	The number of positive binding data associated with each of the 10 most similar MHC alleles with data in the MHCGlobe training set. Ordered by distance to query MHC.
Data matching residue-position of query MHC allele	34	The number of positive binding data in the MHCGlobe training dataset which have a matching amino acid-position identity to each of the 34 residues in the query pseudosequence amino acids.
Data per Distance Bin	8	The number of positive binding data is associated with training data alleles binned by their BLOSUM62 distance score relative to the query pseudosequence. Bins were by 0.1 increment thresholds up to 0.7. Any pair of alleles with a distance score above 0.7 were included in the >0.7 bin.
Total Positive Binding Data	1	The total number of positive binding data in the MHCGlobe training set.

Supplementary Table 4. Descriptions of MHCPerf features. The input features of MHCPerf for a given query MHC were constructed by comparing the query MHC allele pseudosequence to different attributes of the MHCGlobe training set containing MHC binding data. For data counts only the number of positive binding data were utilized.

Preprocessing Peptide-MHC Binding Data

MHC epitopes curated by IEDB were downloaded at https://iedb.org/downloader.php?file_name=doc/mhc_ligand_full_single_file.zip. Instances were filtered to include only those with class I MHC alleles, with a linear peptide epitope and peptide length between 8 to 15 amino acids (i.e., *MHC allele class* = I, *Epitope Object Type* = Linear peptide). IEDB contains both quantitative and qualitative MHC-I epitope data, depending on whether the study used *in vitro* binding affinity assays or elution studies. Quantitative records were identified by *"Quantitative measurement"* !="NA", *dataset_type="BA"* and *Units="nM"*. Qualitative records were identified by *"Quantitative measurement"* ="NA" and *Units!="nM"*. In order to add data that is not included in IEDB, we identified binding records released by MHCFlurry 2.0[16] which were absent from IEDB. The MHCFlurry 2.0 datasets were downloaded from https://data.mendeley.com/datasets/zx3kjzc3yx/3. From the MHCFlurry mass spectrometry dataset, we first removed 52,642 entries corresponding to Class II MHC measurements. Next, records obtained via binding affinity assays, multi-allelic (MA) assays and single-allelic (SA) assays from both IEDB and MHCFlurry data were each filtered for overlap separately. MA records are peptide-MHC binding instances from experiments with cell lines naturally encoding multiple class-I MHC alleles, whereas SA records are derived from cell lines engineered to have a single class-I MHC allele. In the IEDB dataset, SA records are distinguished from MA records by *"Allele Evidence Code*"=*"Single allele present"*. For each data type (BA, SA and MA), MHCFlurry peptide-MHC records were added to the IEDB dataset if absent from the corresponding partition of the IEDB dataset. The MA dataset from MHCFlurry (S1 dataset) does not contain peptide-allele assignments, but rather best allele assignments by multiple pan-MHC binding tools. To determine best assignments to supplement our training dataset, we utilized MHC assignments which agree among all of three previously published methods, NetMHCpan 4.1 EL[11], MHCFlurry 2.0 BA[16] and MixMHCpred 2.0.2[14]. We ran NetMHCpan 4.1 EL to assign the best MHC to each peptide, and assignments for the other two methods were already published as columns within the S1 dataset. MHC allele assignments were in agreement among all three methods for 68,555 peptide-MHC observations out of the 91,581 observed epitopes in multiallelic data, and were added to our curated dataset after confirming the data was not in the IEDB MA dataset.

Further preprocessing steps were taken on the compiled epitope dataset. For quantitative records with multiple observations of a given peptide-MHC pair, the mean binding affinity value was assigned to the peptide-MHC pair. Records were excluded if the difference between maximum and minimum log-transformed values exceeded 0.2, as previously described[33] [i.e., $(1 - \log(\text{nM})/\log(50{,}000)) > 0.2$]. All positive qualitative records were assigned a quantitative affinity value of 100nM and measurement inequality of "<" as performed by O'Donnell *et. al.*[16]. The measurement inequalities (< and >) are used for semi-quantitative and qualitative records so that binding prediction models do not penalize predictions which are within the range of certainty for the associated binding affinity. All qualitative negatives were assigned a very low binding affinity value sampled between 20,000 and 50,000nM to increase diversity among samples during training, and assigned a measurement inequality of ">" to account for the uncertainty in the binding affinity value. Measurement inequalities "<=" and ">=" recorded in the IEDB were mapped to "<" and ">" respectively.
